# Allyl Isothiocyanate Ameliorates Angiogenesis and Inflammation in Dextran Sulfate Sodium-Induced Acute Colitis

**DOI:** 10.1371/journal.pone.0102975

**Published:** 2014-07-22

**Authors:** Munkhtugs Davaatseren, Jin-Taek Hwang, Jae Ho Park, Myung-Sunny Kim, Shuaiyu Wang, Mi Jeong Sung

**Affiliations:** 1 Research Division Emerging Innovative Technology, Korea Food Research Institute, Songnam, Keongki, Republic of Korea; 2 Department of Bioresources and Food Science, Konkuk University, Seoul, Republic of Korea; 3 Institute of Oceanology, Chinese Academy of Sciences, Qingdao, Shandong, China; Massachusetts General Hospital, United States of America

## Abstract

Allyl isothiocyanate (AITC) is a phytochemical found in cruciferous vegetables that has known chemopreventive and chemotherapeutic activities. Thus far, the antiangiogenic activity of AITC has not been reported in *in vivo* studies. Herein, we investigated the effect of AITC on angiogenesis and inflammation in a mouse model of colitis. Experimental colitis was induced in mice by administering 3% dextran sulfate sodium via drinking water. To monitor the activity of AITC in this model, we measured body weight, disease activity indices, histopathological scores, microvascular density, myeloperoxidase activity, F4/80 staining, inducible nitric oxide synthase (iNOS) expression, cyclooxygenase-2 (COX-2) expression, and vascular endothelial growth factor (VEGF)-A/VEGF receptor 2 (VEGFR2) expression in the mice. We found that AITC-treated mice showed less weight loss, fewer clinical signs of colitis, and longer colons than vehicle-treated mice. AITC treatment also significantly lessened the disruption of colonic architecture that is normally associated with colitis and repressed the microvascularization response. Further, AITC treatment reduced both leukocyte recruitment and macrophage infiltration into the inflamed colon, and the mechanism these activities involved repressing iNOS and COX-2 expression. Finally, AITC attenuated the expression of VEGF-A and VEGFR2. Thus, AITC may have potential application in treating conditions marked by inflammatory-driven angiogenesis and mucosal inflammation.

## Introduction

Inflammatory bowel disease (IBD) is a chronic inflammatory disorder, of which there are two major types—Crohn's disease and ulcerative colitis. Although the etiopathogenesis of IBD has not been definitively elucidated, it is widely accepted that both Crohn's disease and ulcerative colitis result from a complex interaction between genetic, environmental, and intestinal immune factors [1]–[3]. IBD is also known to be associated with extensive edema-induced tissue injury and remodeling, inflammatory cell infiltration, numerical or functional alteration of immune cell subpopulations, loss of epithelial integrity, and increased angiogenesis. These features are thought to contribute to the pathophysiology and development of IBD through diverse molecular mechanisms that can involve several cell types and mediators [4], [5].

Angiogenesis is now known to be intimately involved in a number of biological processes, including growth, development, and tissue repair [6]. In some disease states such as tumorigenesis and chronic inflammation, angiogenesis becomes pathologic and therefore represents a potential therapeutic target. There is considerable evidence that abnormal angiogenesis is a key pathology of many chronic inflammatory diseases like rheumatoid arthritis, atherosclerosis, and diabetic retinopathy [7], [8]. Recent human and animal studies revealed that angiogenesis also plays a crucial role in IBD [9]–[11].

Vascular endothelial growth factor-A (VEGF-A) is an important angiogenic mitogen that has become the primary target for antiangiogenic drug therapies. VEGF-A is a fundamental mediator of pathologic angiogenesis in several inflammatory disorders, including neoplasia, chronic inflammation, and IBD [8], [12]. The microvasculature in IBD likely contributes to bowel inflammation by affecting innate immunity, leukocyte infiltration, coagulation, and vascular permeability [13]–[15]. A number of studies have demonstrated that direct inhibition of VEGF or its primary receptor VEGFR2 decreases inflammation and can potentially lessen colonic tissue damage by reducing vascularization or by altering vessel permeability [16], [17]. In human and experimentally modeled IBD, the reduced vascularization induced by VEGF decreases the delivery of inflammatory cells to colonic injury sites, thereby disrupting the damaging inflammation-angiogenesis cycle.

One class of compounds that may be useful for treating IBD is the isothiocyanates (ITCs), which are found as thioglucoside conjugates known as glucosinolates in many cruciferous vegetables like cabbage, cauliflower, and Brussels sprouts. Many ITCs can prevent the development of chemically induced tumors in a variety of animals [18]–[20]. Allyl isothiocyanate (AITC) is a cancer chemopreventive phytochemical agent found in many dietary sources. Previous studies have indicated that AITC can inhibit the growth of various types of tumor cells by causing cell cycle arrest and inducing apoptosis [21]–[23]. AITC has also been shown to have proapoptotic and antiangiogenic activity against ascites tumor cells in mice [24]. The tumor-specific angiogenic activity of AITC appears to derive in part from repressing the production of nitric oxide (NO) and tumor necrosis factor-alpha (TNF-alpha) [25]. Additionally, AITC inhibits endothelial cell differentiation and proinflammatory cytokine production during angiogenesis [26]. Recent studies even suggest that AITC inhibits metastasis of HT29 colorectal cells [27]. Until now, however, there has been no report of an antiangiogenic effect of AITC in chronic inflammatory conditions or IBD. Based on these activities, it is reasonable to propose that the antiangiogenic activity of AITC could also significantly impact inflammation and disease pathology associated with various chronic inflammatory conditions. To evaluate this possibility, we have evaluated the prophylactic properties of AITC against intestinal inflammation and angiogenesis in a mouse model of DSS-induced colitis.

## Materials and Methods

### Experimental colitis model

All animal procedures were approved by the Institutional Animal Care and Use Committee of the Korea Food Research Institute. Male, 8-week-old C57BL/6 mice were obtained from Charles River Korea (Seoul, Korea) and housed in the Korea Food Research Institute (KFRI) at a temperature of 22–26°C under a 12 h light/12 h dark cycle with free access to food and tap water. Mice were allowed to adapt to their food and environment for 1 week before the start of the experiment. The animals were matched by body weight and then randomly assigned to 4 groups of 8 mice each. Experimental colitis was established as previously described [17] by adding 3% w/v dextran sulfate sodium (DSS, MW ¼ 36–50 kDa; MP Biochemicals, Aurora, OH) to the drinking water for 7 days. Mice in group 1, the control group, received tap water without DSS. Concurrent with DSS treatment, the remaining three groups were dosed as follows: group 2, dosed with DSS and corn oil vehicle (DSS); group 3, dosed daily with 3% DSS and 10 mg/kg AITC suspended in corn oil (D+A10); and group 4, dosed daily with 3% DSS and 25 mg/kg AITC suspended in corn oil (D+A25). On day 7, mice were sacrificed by cardiac puncture under ketamine/xylazine anesthesia. Histological colon samples were isolated and fixed in cold 4% phosphate-buffered formalin or frozen at −20°C for myeloperoxidase (MPO) and western blotting analysis.

### Body weight changes and disease activity index

Body weights were measured daily beginning 1 day prior to DSS administration and continuing for 7 days until sacrifice. In this model, mice typically lose approximately 20% of their body weight by day 7 of continuous exposure to 3% DSS in their drinking water. Evidence for onset of erosive distal colitis is observed by day 3 or 4 and is characterized by progressive weight loss, diarrhea, occult blood, leukocyte infiltration, colon shortening, loss of intestinal epithelial barrier, and histopathological changes in the colon structure. For daily assessment of disease progression, treatment groups were assigned a daily clinical disease activity index (DAI) as previously described [28], [29]. Briefly, DAI was determined by averaging weight loss, stool consistency, and bleeding scores. Weight loss scores were assigned as follows: <1% weight loss, 0; 1–5% weight loss, 1; 5–10% weight loss, 2; 10–15% weight loss, 3; and >15% weight loss, 4. Stool consistency scores were assigned as follows: normal, 0; loose stools, 2; and diarrhea, 4. Bleeding scores were assigned as follows: negative, 0; positive, 2; and gross bleeding, 4.

### Histopathological colitis scoring

Formalin-fixed colon sections were stained with hematoxylin and eosin (H&E) and then analyzed under light microscopy at 40× magnification with at least 4 fields assigned a score for colitis severity by an examiner without prior knowledge of experimental procedures [30]. Assessment included noting of edema, extent of injury, leukocyte infiltration, crypt abscesses, and the loss of goblet cells. To assign a severity score, 2 mutually blinded observers graded the tissues and assigned scores for several parameters as follows: inflammation severity (0, none; 1, slight; 2, moderate; and 3, severe), the extent of injury (0, none; 1, mucosal; 2, mucosal and submucosal; and 3, transmural), crypt damage (0, none; 1, basal third damaged; 2, basal two-thirds damaged; 3, only surface epithelium intact; and 4, loss of entire crypt and epithelium). The scores for each parameter were multiplied by an extent score that reflected the percentage of each section that had a given characteristic as follows: 1, 0–25%; 2, 26–50%; 3, 51–75%; and 4, 76–100%. Using this scoring system, the minimum and maximum possible scores for each sample were 0 and 40, respectively.

### Immunofluorescence analysis

Colon tissues were cut into 10 µm sections. Slides were incubated with hamster anti-mouse CD31 monoclonal antibody (Chemicon, Temecula, CA) and rat anti-mouse F4/80 antibody (Invitrogen, Carlsbad, CA) overnight at 4°C. Cy3-conjugated anti-hamster and FITC-conjugated anti-rat immunoglobulin G (IgG) antibodies were used as the secondary antibody for visualization. Samples were then visualized using a Nikon Eclipse T*i* confocal microscope (Thornwood, NY). The density of vascularization in each colon section was quantified as a percentage of tissue area immunopositive for CD31 at a magnification of 200× in 5 regions, each amounting to a 0.21-mm^2^ area. The number of F4/80 cells in colon sections was measured by F4/80 staining.

### Western blot analysis

Immunoblotting was performed as described previously [17]. Each frozen colon tissue specimen (10–20 mg) was homogenized in phosphate-buffered saline (PBS) containing a protease inhibitor cocktail (Calbiochem, San Diego, CA), and the total protein concentration was quantitated. Proteins samples (50 µg of total protein per lane) were mixed with sample buffer, boiled for 10 min, separated by sodium dodecyl sulfate-polyacrylamide (10%) gel electrophoresis under denaturing conditions, and electroblotted onto nitrocellulose membranes. The membranes were incubated overnight at 4°C with anti-VEGF-A and antibodies for inducible (iNOS, Santa Cruz Biotechnology, Santa Cruz, California), VEGFR-2 (GeneTex, San Antonio, TX), and cyclooxygenase-2 (COX-2, Cayman Chemical, San Antonio, TX). The membranes were stripped and then re-blotted with anti-actin (dilution, 1∶2,000; Sigma, St. Louis, MO) to verify equal protein loading in each lane. Experiments were repeated 3 times.

### Myeloperoxidase activity assay

Neutrophil infiltration into the colon was assessed indirectly by measuring myeloperosidase (MPO) activity as described previously with slight modifications [17]. Colon segments (20–30 mg) were frozen in liquid nitrogen, crushed, and freeze-thawed three times in hexadecyltrimethylammonium buffer (0.5% w/v in water, Sigma, St. Louis, MO). Samples were then sonicated for 10 s at 50% of maximum power and cleared by centrifugation at 10,000×*g* before measuring MPO activity in the supernatants using 0.1% *o*-dianisidine as substrate. The change in absorbance 460 nm was measured using a SpectraMax M2 Microplate Reader (Molecular Devices, Sunnyvale, CA). MPO activity was calculated as the amount of enzyme necessary to produce a change in absorbance of 1.0 unit per minute per gram of tissue (wet weight).

### Statistical analysis

All data are expressed as a mean ± standard deviation. Analysis of variance (ANOVA) was used to compare data between groups. A value of *p*<0.05 was considered to indicate a statistically significant difference.

## Results

### AITC ameliorates the symptoms of DSS-induced colitis in mice

**Figure 1:**
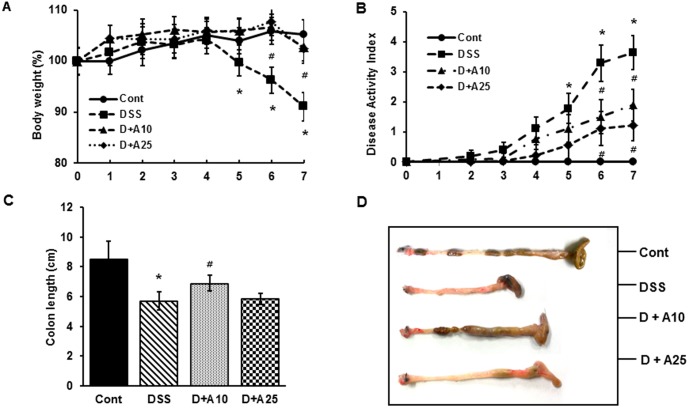
Effect of AITC on clinical symptoms in a mouse colitis model. (A) Changes in body weights and (B) clinical scores of AITC–treated mice and control mice administered 3% DSS were monitored every day. Body weight values are expressed as a percentage of the starting body weight. (C) Colons were excised from mice and their lengths were measured 7 days after initiating DSS administration. (D) Macroscopic features of the colons. Control group (without DSS; Cont), 3% DSS administration group (DSS), 3% DSS with AITC at 10 mg/kg per day (D+A10), 3% DSS with AITC at 25 mg/kg per day (D+A25). Data shown are an aggregate of 3 independent experiments and are expressed as mean ± SD (*n* = 8 per group). * *p*<0.05, DSS versus Cont; ^#^ *p*<0.05, D+A10 and D+A25 versus DSS.

To examine the potential of AITC for treating IBD symptoms we evaluated its activity in a DSS-induced colitis model in mice. Colitis was induced in C57BL/6 mice by inclusion of 3% DSS in their drinking water. Concurrent with DSS treatment, mice were treated orally with either the corn oil vehicle or AITC at 10 or 25 mg/kg for 7 days. Mice in the control group that only received vehicle (DSS) developed the typical clinical signs of colitis, including weight loss, diarrhea, and rectal bleeding. The body weights of DSS-treated mice were significantly decreased compared to mice that did not receive DSS. Mice that received either 10 or 25 mg/kg AITC concurrent with DSS treatment (D+A10 and D+A25, Figure 1) lost less weight than did DSS-treated mice. AITC treatment also significantly lowered the DAI index compared to the DSS group (Figure 1B). Shortening of the colon is also symptomatic of DSS-induced colitis in mice, as evidenced by the shorter colon length in DSS-treated mice compared to non-treated mice. AITC treatment effectively reduced the shortening of the colon (Figure 1C and D).

### AITC reduces microscopic colon damage during DSS-induced colitis in mice

**Figure 2:**
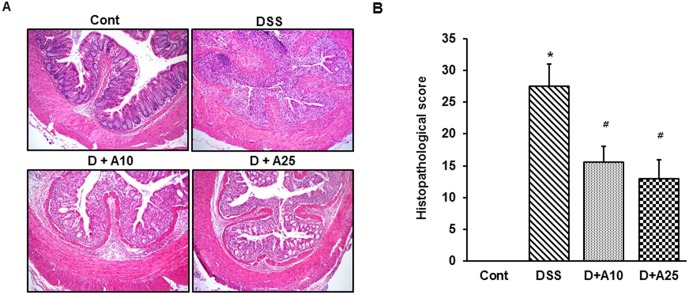
Effect of AITC on histopathological signs in a mouse colitis model. (A) Colons were excised 7 days after initiating DSS administration, and were then sectioned and stained with hematoxylin and eosin. Control group (without DSS; Cont), 3% DSS administration group (DSS), 3% DSS with AITC at 10 mg/kg per day (D+A10), 3% DSS with AITC at 25 mg/kg per day (D+A25). Original magnification: 40×. (B) Histopathological scores of the analyzed slides. Bars represent the mean ± SD from 3 slides per mouse. * *p*<0.05, DSS versus Cont; ^#^ *p*<0.05, D+A10 and D+A25 versus DSS.

To investigate the cellular effects of AITC in DSS-induced colitis, we carried out H&E staining for colonic sections from each treatment group. As expected, oral administration of DSS induced inflammatory changes in colonic architecture that are typical of colitis, including ulceration, crypt dilation, and goblet cell depletion as well as mixed cell infiltration by macrophages and lymphocytes. Treatment with AITC at 10 mg/kg and 25 mg/kg significantly decreased cell infiltration and repressed mucosal injury and edema (D+A10 and D+A25, Figure 2A). Furthermore, H&E-stained colonic sections were histologically scored under light microscopy to assess intestinal inflammatory status. Compared to untreated mice, DSS-treated mice showed significantly increased histological scoring of inflammation, whereas both AITC doses significantly decreased inflammation (Figure 2B).

### AITC decreased angiogenesis during colitis

**Figure 3:**
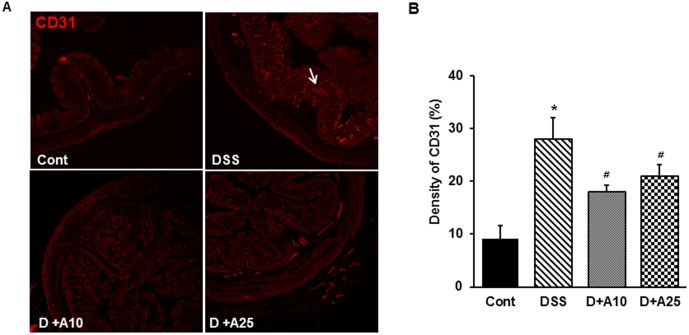
Effect of AITC on angiogenesis in a mouse colitis model. (A) Colonic microvasculature stained for endothelial cells (200× magnification) using the CD31 antibody. Control group (without DSS; Cont), 3% DSS administration group (DSS), 3% DSS with AITC at 10 mg/kg per day (D+A10), 3% DSS with AITC at 25 mg/kg per day (D+A25). (B) Analysis of the area densities of blood vessels. Bars represent the mean ± SD from 3 slides per mouse. * *p*<0.05, DSS versus Cont; ^#^ *p*<0.05, D+A10 and D+A25 versus DSS.

To investigate the effects of AITC on angiogenesis, we measured the expression of CD31, a blood vessel endothelial cell marker, and we quantified the microvascular density in colon sections (Figure 3). Compared to untreated mice, DSS-treated mice had a significantly increased microvascular density, whereas AITC treatment significantly decreased number of mucosal vessels.

### AITC decreased inflammatory cell infiltration during colitis

**Figure 4:**
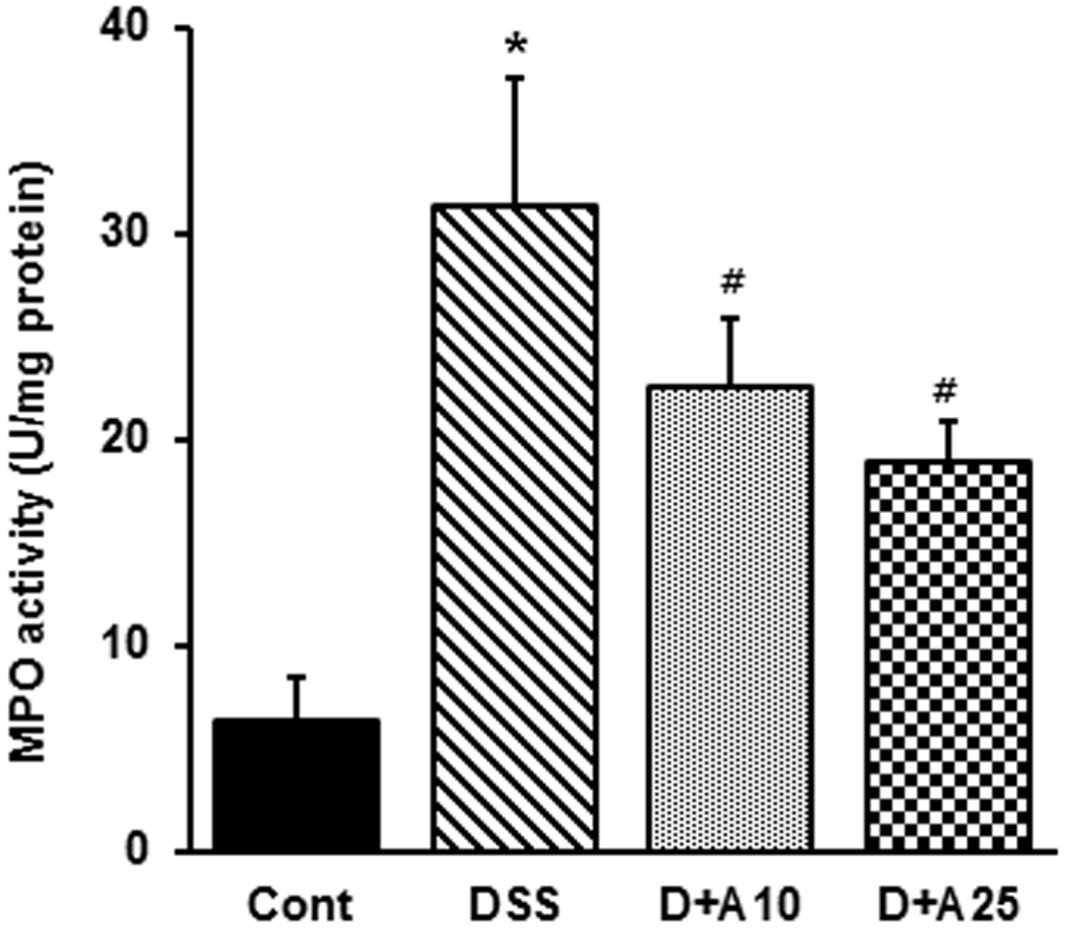
Effect of AITC on MPO activity in a mouse colitis model. Control group (without DSS; Cont), 3% DSS administration group (DSS), 3% DSS with AITC at 10 mg/kg per day (D+A10), 3% DSS with AITC at 25 mg/kg per day (D+A25). Data shown represent the aggregate of 3 independent experiments, expressed as mean ± SD (*n* = 8 per group). * *p*<0.05, DSS versus Cont; ^#^ *p*<0.05, D+A 10 and D+A 25 versus DSS.

**Figure 5:**
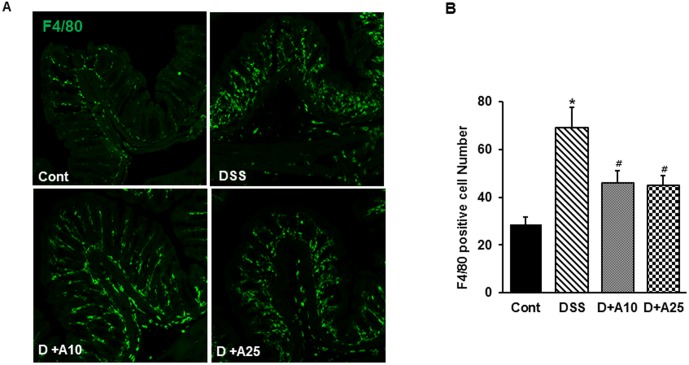
Effect of AITC on F4/80 positive cell number in a mouse colitis model. (A) Colon stained for macrophage (200× magnification) using the F4/80 antibody. Control group (without DSS; Cont), 3% DSS administration group (DSS), 3% DSS with AITC at 10 mg/kg per day (D+A10), 3% DSS with AITC at 25 mg/kg per day (D+A25). (B). Analysis of the F4/80 positive cell number. Bars represent the mean ± SD from 3 slides per mouse. * *p*<0.05, DSS versus Cont; ^#^ *p*<0.05, D+A10 and D+A25 versus DSS.

To determine whether AITC affects infiltration of inflammatory cells in DSS-induced colitis we measured MPO activity, and number of F4/80 positive cell. As such, MPO levels generally correlate with the level of neutrophil infiltration in tissues. In our model, MPO activity was markedly higher in DSS-treated mice than in untreated mice, and AITC significantly reduced MPO activity (Figure 4). Consistent with this result, F4/80 positive cell levels were significantly higher after DSS treatment but reduced by concurrent AITC treatment (Figure 5).

### AITC decreased iNOS and COX-2 expression during colitis

**Figure 6:**
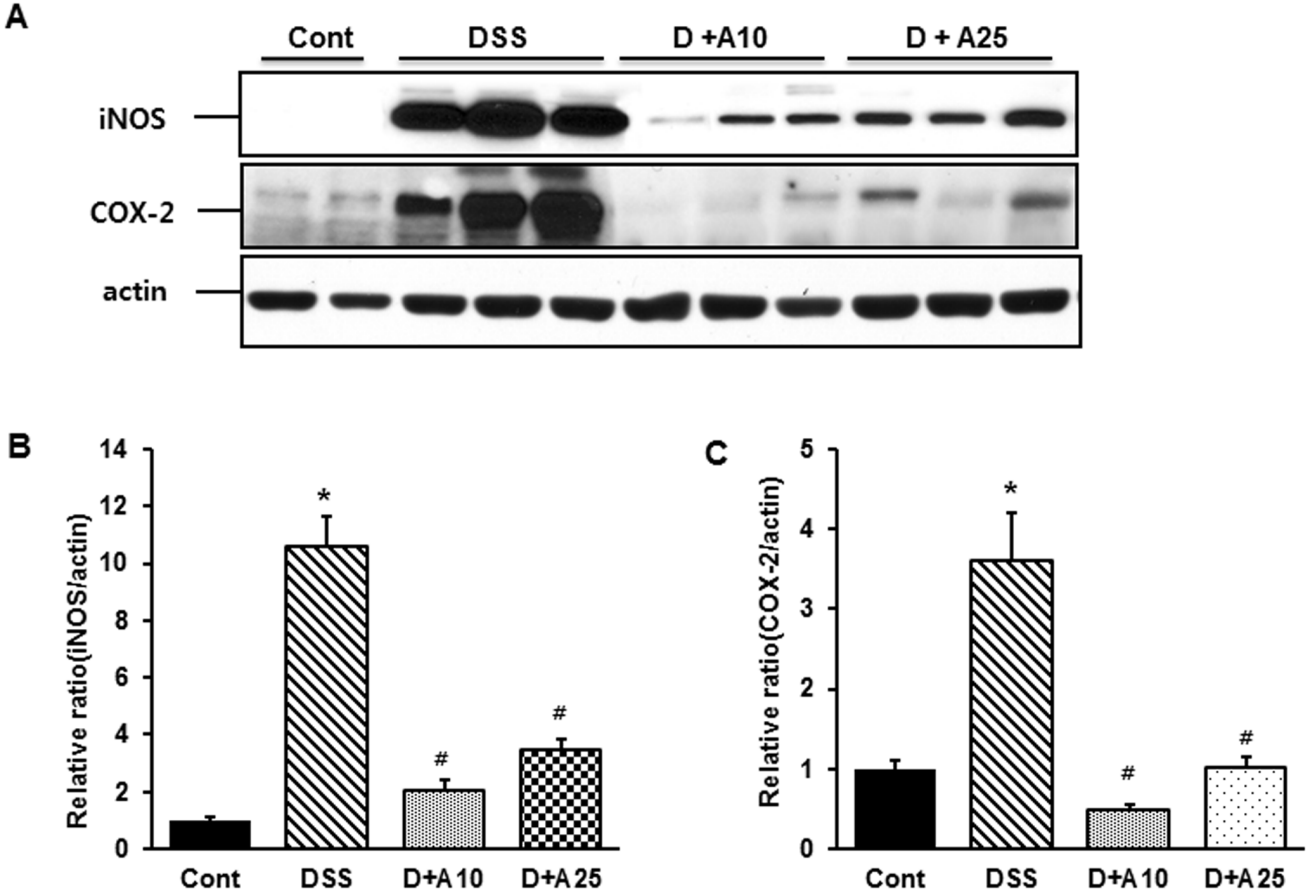
Effect of AITC on expression of iNOS and COX-2 in a mouse colitis model. (A) iNOS and COX-2 expression were determined by immunoblotting of colon samples from the control group (without DSS; Cont), 3% DSS administration group (DSS), 3% DSS with AITC at 10 mg/kg per day (D+A10), 3% DSS with AITC at 25 mg/kg per day (D+A25). β-Actin was used as an internal control. (B) and (C) Densitometric analyses are presented as the relative ratio of each protein to actin. The ratio relative to the control is arbitrarily presented as 1. Bars represent the mean ± SD from 3 experiments. * *p*<0.05, DSS versus Cont; ^#^ *p*<0.05, D+A10 and D+A25 versus DSS.

Western blot analysis specific for iNOS and COX-2 was used to assess whether AITC directly reduced inflammation during DSS-induced colitis. Significant levels of both proteins were detected in all groups, with expression clearly stimulated in the DSS-treated group (Figure 6). Notably, both dose levels of AITC repressed iNOS and COX-2 expression significantly (Figure 6A and B).

### AITC decreased VEGF-A and VEGF-R2 expression during colitis

**Figure 7:**
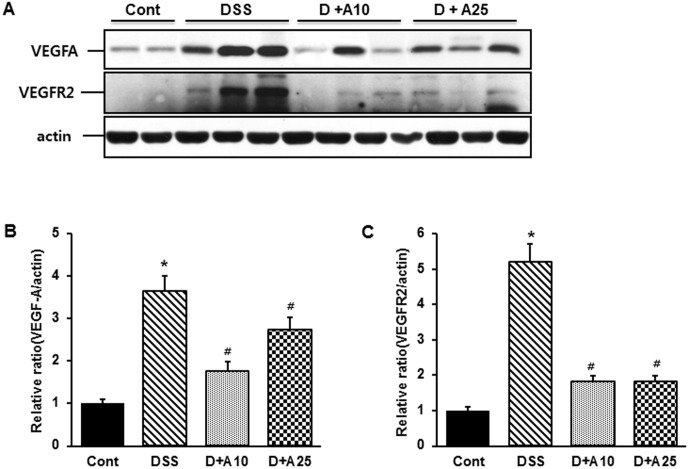
Effect of AITC on expression of VEGF-A and VEGFR2 in a mouse colitis model. (A) VDGF-A and VEGFR2 expression were determined by immunoblotting in colon samples from the control group (without DSS; Cont), 3% DSS administration group (DSS), 3% DSS with AITC at 10 mg/kg per day (D+A10), 3% DSS with AITC at 25 mg/kg per day (D+A25). β-Actin was used as an internal control. (B) and (C) Densitometric analyses are presented as the relative ratio of each protein to actin. The ratio relative to the control is arbitrarily presented as 1. Bars represent the mean ± SD from 3 experiments. * *p*<0.05, DSS versus Cont; ^#^ *o*<0.05, D+A10 and D+A25 versus DSS.

VEGF-A is a very well characterized and fundamental mediator of pathogenic angiogenesis [31]. As such, we considered it a marker for measuring the effect of AITC on angiogenesis during colitis. We therefore used western blot analysis to evaluate the levels of VEGF-A and VEGF-R2 in colonic sections. As expected, DSS-treatment increased VEGF-A and VEGF-R2 levels compared to the control group, whereas concurrent treatment with AITC demonstrably decreased VEGF-A and VEGF-R2 expression (Figure 7).

## Discussion

Several previous studies have shown that the dietary isothiocyanate AITC can significantly inhibit the growth of several types of chemically induced tumors in animal models [21]-[23], [32]. In addition, it has been reported that AITC can affect tumor-associated angiogenesis [24]–[27]. To date, however, the effects of AITC on immune functions, especially angiogenesis, have not been fully characterized. Herein we have demonstrated that AITC exhibits prophylactic activity by reducing inflammation-driven angiogenesis. This could have beneficial applications in the treatment of IBD.

IBD includes several idiopathic chronic inflammatory disorders of the intestine and/or colon in which patients suffer from rectal bleeding, severe diarrhea, abdominal pain, and loss of body weight. Although the processes underlying the onset of IBD are poorly understood, the pathologies defining IBD have been extensively characterized. IBD-afflicted colons are characterized by a large number of leukocytes in the intestinal and/or colonic interstitium that result in granulomatous inflammation. Coincident with inflammatory cell infiltration is extensive transmural injury, including edema, loss of goblet cells, crypt cell hyperplasia, erosions, and ulceration [33]. Several animal models of IBD have been developed for evaluating potential therapeutic agents. Among these, a mouse model in which colitis is established by DSS treatment is perhaps the most widely utilized. The number of genes involved in angiogenesis in this model has been shown to be quite similar to those in human ulcerative colitis [34], [35], suggesting that this model can be useful for estimating the therapeutic potential of test agents for human treatment. In the present study, DSS-treated mice show symptoms characteristic of ulcerative colitis, including weight loss, shortening of the colon, and an increased DAI score. Histopathological analysis also revealed that DSS treatment results in extensive interruption of the epithelial surface, with submucosal edema and inflammatory cell infiltration that consisted of dense lymphoid aggregates devoid of germinal centers. Treatment with AITC concurrent with DSS treatment significantly ameliorates all of the symptoms associated with colitis. AITC-treated mice showed less weight loss and lower DAI scores than mice treated with only DSS (Figure 1). Mice treated with AITC also showed attenuated tissue injury; their colons retained a more intact epithelial surface with little inflammation (Figure 2). Collectively, these results suggest that AITC does attenuate DSS-induced colitis injury.

Angiogenesis plays an important role in many neoplastic and chronic inflammatory disorders, including IBD [36]. Active microvessel changes associated with angiogenesis are fundamental to the pathogenesis of IBD and have been observed in acutely inflamed colon tissues from patients with ulcerative colitis and Crohn's disease. Recent evidence in human and experimental colitis has further supported the important role of microvascularization in IBD [33], [34]. Microscopic imaging is the most utilized approach for quantifying vasculature in normal and pathological tissues [37]–[38], including evaluations of microvascularization associated with IBD. In the latter cases, PECAM-1/CD31 staining revealed a significant vessel density increase at the mucosal and submucosal tissue layers in IBD patients [16]–[17], [39]. Therefore, we utilized CD31 staining to assess microvascular density in colonic sections from mice. We found that AITC treatment significantly decreased the level of CD31 compared to mice treated only with DSS and vehicle (Figure 3). These results confirm that AITC represses the microvascular growth associated with angiogenesis in DSS-induced colitis.

Mediators of chronic inflammation, both cellular (e.g., leukocytes and platelets) and biochemical (cytokines and chemokines), are potent stimuli for angiogenesis [40], [41]. The immune pathogenesis of IBD is associated with an increase of chronic inflammatory mediators, including infiltration of neutrophils, activation of macrophages, and unregulated production of proinflammatory molecules in the colon. Several recent studies have also shown that neutrophil and macrophage infiltration may be triggered by angiogenesis and that these events are causally associated [40]. Correspondingly, the neutrophils and macrophages that infiltrate the inflamed gut provide many of the cytokines, growth factors, proteolytic enzymes, and oxidants that contribute to injury and inflammatory angiogenesis. In addition, activated leukocytes stimulate the expression of a number of inflammatory mediators such as iNOS and COX-2 [42]. Our results demonstrate that, during DSS-induced colitis in mice, MPO activity and F4/80 cell numbers were significantly increased, and ATIC treatment markedly attenuated these responses (Figures 5 and 6). Likewise, AITC treatment markedly reduced iNOS and COX-2 expression (Figure 7). Collectively, these data suggest that AITC can effectively dampen the proinflammatory colitis response in mice.

The expression of several growth factors and their receptors is also known to be stimulated as part of the IBD angiogenesis process. For example, VEGF is a major proangiogenic factor that appears to be essential in the development of IBD and experimentally induced colitis. Consistent with this role, blocking the expression of VEGF-A or its receptors has been demonstrated to reduce the severity of colitis [43]–[45]. Consequently, VEGF-A is considered a useful target for developing new IBD therapies. In the present study, we demonstrated that the expressions of VEGF-A and VEGFR2 are significantly increased in mice with DSS-induced colitis. Importantly, AITC treatment repressed the expression of VEGF-A and VEGFR2, revealing another mechanism by which AITC can suppress colitic inflammation and angiogenesis (Figure 4).

In this study, we demonstrated that AITC significantly attenuated the clinical symptoms and histological features characteristic of IBD in mice with experimentally induced colitis. AITC reduced the infiltration of inflammatory cells through a mechanism associated with inhibition of inflammatory mediators. AITC also significantly reduced VEGF-A and VEGFR2 expression, indicating that the mechanism of action may involve a repression of proangiogenesis. Taken together, our results suggest that AITC has significant potential as a developmental preventive for conditions characterized by mucosal inflammation and associated angiogenesis.
